# Prognostic value of intracranial vascular tortuosity in thrombectomy for distal vessel occlusion

**DOI:** 10.1093/esj/23969873251350124

**Published:** 2026-01-01

**Authors:** Pere Canals, Alvaro García-Tornel, Giulio Maria Fiore, Marc Rodrigo-Gisbert, Blanca Sastre, Jordi Mayol, Marc Ribo

**Affiliations:** Stroke Unit, Neurology, Hospital Vall d’Hebron, Barcelona, Spain; Department of Radiology, Stanford University, Stanford, CA, USA; Stroke Unit, Neurology, Hospital Vall d’Hebron, Barcelona, Spain; Stroke Unit, Neurology, Hospital Vall d’Hebron, Barcelona, Spain; Stroke Unit, Neurology, Hospital Vall d’Hebron, Barcelona, Spain; Departament de Medicina, Universitat Autònoma de Barcelona, Barcelona, Spain; Stroke Unit, Neurology, Hospital Vall d’Hebron, Barcelona, Spain; Stroke Unit, Neurology, Hospital Vall d’Hebron, Barcelona, Spain; Departament de Medicina, Universitat Autònoma de Barcelona, Barcelona, Spain; Stroke Unit, Neurology, Hospital Vall d’Hebron, Barcelona, Spain; Departament de Medicina, Universitat Autònoma de Barcelona, Barcelona, Spain

**Keywords:** Stroke, thrombectomy, vascular tortuosity, CT angiography, neuroimaging, artery, catheter, ischemic stroke, imaging, distal vessel occlusion

## Abstract

**Introduction:**

Neutral results from trials assessing mechanical thrombectomy (MT) for medium/distal vessel occlusions (MDVO) suggest the need for better selection criteria in these patients. Tortuous vascular anatomies may negatively influence MT efficacy and safety.

**Patients and methods:**

Consecutive patients with middle cerebral artery (MCA)-MDVO (M2/M3) who underwent MT at our center between January 2017 and September 2024 were included. Baseline CTAs were semi-automatically analyzed using an in-house vascular analysis framework. The internal carotid artery (ICA) tortuosity index (TI) and anatomical features of the MCA were extracted. Logistic regression adjusted for intravenous thrombolysis administration and onset-to-puncture time evaluated associations of anatomical features with treatment efficacy and safety endpoints. Primary endpoints were complete recanalization (final eTICI 2c/3) and symptomatic intracranial hemorrhage (sICH).

**Results:**

213 patients (81 years IQR 72–87, 51.2% female) were included. MCA bending length (aOR 0.48 [95%CI 0.27–0.86], *p* = 0.013), MCA-TI (aOR 0.77 [0.60–0.98], *p* = 0.032) and ICA-TI (aOR 0.59 [0.36–0.96], *p* = 0.034) were associated with lower probability of complete recanalization. ICA-TI (aOR 0.51 [0.31–0.84], *p* = 0.008) and mean MCA diameter (aOR 0.34 [0.13–0.90], *p* = 0.030) correlated with decreased odds of first-pass recanalization. Large mean MCA diameter was associated with lower likelihood of excellent functional outcome (aOR 0.30 [0.09–0.96], *p* = 0.042). Regarding safety endpoints, larger diameter at occlusion was associated with sICH (aOR 4.04 [1.03–15.87], *p* = 0.046), while MCA bending length (aOR 2.47 [1.24–4.92], *p* = 0.010) was linked to subarachnoid hemorrhage.

**Discussion:**

Automatic evaluation of anatomical vascular features may predict safety and efficacy of MT in stroke patients with MCA-MDVO. The value of these features as inclusion criteria for future MCA-MDVO clinical trials should be explored.

**Conclusion:**

Intracranial vascular tortuosity is associated to poor thrombectomy outcomes in patients with MDVO.

## Graphical Abstract

**Graphical abstract:**
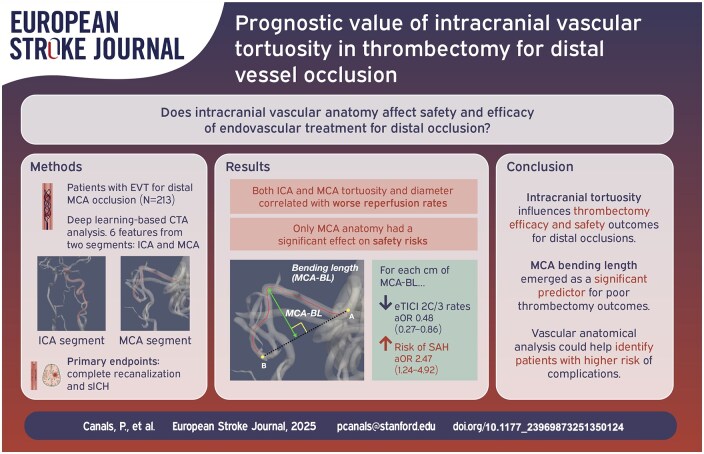

## Introduction

In the last decade, successive positive clinical trials expanded the indication of MT for acute large vessel occlusion (LVO) strokes to patients admitted in the extended window^1,2^ or with a large ischemic core on initial neuroimage,^3–5^ gradually broadening the target population who may benefit from the procedure.^6^ One of the unconquered frontiers in the indications of MT is the presence of a medium/distal vessel occlusions (MDVO); if proven beneficial, the procedure could improve clinical outcomes in up to 25%–40% of all acute ischemic strokes.^7^ Unfortunately, recently published trials evaluating MT in MDVO strokes have presented neutral results, failing to show benefit of MT over best medical treatment.^8,9^ On one hand, these results clarify the contradictory observations found in previous retrospective observational studies,^10,11^ but also suggest that improved selection criteria, devices and techniques, more specific for MDVO should be explored.

There may be multiple causes explaining the neutral results of the MT trials in MDVO. One differential aspect of MDVOs compared to LVOs is the potential higher influence of intracranial vascular anatomy on MT outcomes. While the intracranial vascular segments that need to be navigated to reach an LVO are generally short, homogeneous and relatively straight, there might be a high heterogeneity within MDVO cases depending on the occlusion location and patient-specific vascular anatomy, including some cases with long and narrow segments, severe tortuosity or both. These characteristics may condition the ability of the neurointerventionalist to safely navigate catheters, position devices or effectively perform MT passes in MDVO patients,^12,13^ resulting in reduced rates of recanalization success and increased safety concerns in selected cases.^7,14^ Features like arterial diameter or vessel curves have not been accounted for in the recently published neutral MDVO trials for patient selection. Identifying anatomical predictors of poor MT outcomes based on fast, automated analysis of baseline neuroimaging may be a first step toward an advanced, individualized triage of patients with a higher likelihood of treatment success.

While several studies have shown the influence of extracranial cervical artery morphology on MT success,^15,16^ focusing primarily on intracranial access, few have quantitatively assessed the impact of intracranial vascular features on MT recanalization rates^12^ or safety,^17^ and even fewer have focused specifically on patients with MDVO where these features may potentially have a higher impact as compared to LVO.^18^

In this study, we aimed to analyze the influence of anatomical descriptors of cervical and intracranial arteries on MT outcomes, namely recanalization success and hemorrhagic transformation (HT), among patients with a MDVO of the middle cerebral artery (MCA). For this purpose we used Arterial, a previously validated in-house academic framework designed for fast vascular analysis of baseline CT angiography (CTA),^19^ to create a semi-automatic processing pipeline for robust and replicable feature extraction of the intracranial and cervical arteries.

## Methods

This study adhered to the STROBE (Strengthening the Reporting of Observational Studies in Epidemiology) guidelines as an observational, cross-sectional study. Ethics approval was obtained from the Comité de Ética de Investigación con Medicamentos (CEIm), the local institutional review board of Hospital Vall d’Hebron (Barcelona, Spain; project reference: PR(AG)484/2021). The need for informed consent of the included patients was waived.

### Study design and population

**Figure 1.**
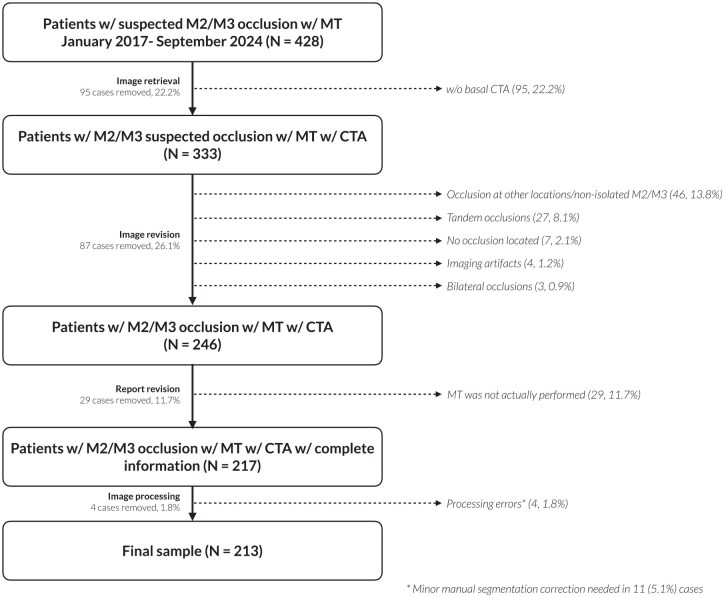
Inclusion chart for the study population. MT: mechanical thrombectomy.

This was a single-center, retrospective study based on a prospectively maintained database where all stroke patients treated with MT are included. Patients who presented evidence of MDVO in the M2 or M3 segments of the MCA on baseline CTA and were treated with MT between January 2017 to September 2024 at our comprehensive stroke center were included. Exclusion criteria were multiple simultaneous occlusions, tandem extra-intracranial occlusions, non-isolated M2/M3 occlusions, imaging artifacts, or unavailability of baseline CTA (e.g. patients following direct transfer to angiosuite protocol). Patients that, despite a qualifying occlusion did not undergo at least one thrombectomy pass, or were wrongfully registered as patients who underwent MT but did only actually undergo a diagnostic angiography, were also excluded upon report revision. Finally, patients for whom image processing errors in feature extraction could not be corrected due to insufficient CTA quality (*n* = 4, 1.8%) were also excluded. Manual correction of automated segmentation was applied in 11 patients (5.1%) to ensure good quality of measurements. An inclusion chart can be found in Figure 1.

### Distal MCA occlusion definition and MT procedure

The definition of distal occlusion employed within this study closely resembles the definition used in the ESCAPE-MeVO trial for MCA occlusions.^9,20^ MDVOs located beyond the main M1 bifurcation were considered proximal M2 if the occlusion was located within 1 cm from the bifurcation. MDVO beyond 1 cm of the main M1 bifurcation were considered distal M2. MDVOs beyond the circular sulcus of the insula were considered M3 occlusions. As opposed to the DISTAL trial inclusion criteria, in order to avoid using subjective criteria, we did not exclude patients according to M2 branch dominance.^8^

Following routine practice at our center, MT procedures were performed via femoral arterial access as the default approach. A 6F 90 cm long sheath is introduced into the vascular access, and supraaortic trunk cannulation is typically achieved using 125 cm long Simmons 2 or Vertebral catheters. After advancing the long sheath to the level of the ICA, interventionalists generally performed the procedure with the combined approach using a distal access catheter together with a stent retriever. Setup for subsequent retrieval attempts was left to operator discretion.

### Study endpoints

We defined the primary efficacy endpoint as complete recanalization, that is, achieving an extended thrombolysis in cerebral infarction score (eTICI) 2c/3 at the end of treatment. Secondary efficacy endpoints included first pass effect (FPE), defined as complete recanalization (eTICI 2c/3) at first thrombectomy pass, successful recanalization (final eTICI 2b/3) and excellent long-term functional outcome as defined by a modified Rankin Scale (mRS) score of 0 or 1 at 3 months. While more clinically relevant, excellent outcome was relegated to a secondary outcome as it was hypothesized that vascular anatomy may present a stronger effect over immediate thrombectomy outcomes, such as reperfusion. Long-term clinical outcomes are typically influenced by a broad interplay of complementary factors, some of which were not considered in this analysis.

The primary safety endpoint was symptomatic intracranial hemorrhage (sICH), defined as intracranial bleeding detected in the 24h follow-up neuroimaging leading to neurological deterioration as an increase in the National Institute of Health Stroke Scale (NIHSS) ⩾4, or deterioration leading to intubation or craniotomy within 7 days, without additional explanation for the worsening.^21^ Secondary safety endpoints included isolated subarachnoid hemorrhage (SAH), parenchymal hematoma type 2 (PH2), as defined by the Heidelberg bleeding classification,^21^ or either SAH or PH2 (severe HT) linked to the MT procedure. We focused on both SAH and/or PH2 post-MT as, in contrast with other types of intracranial hemorrhages of types HI1, HI2 or PH1, these have been independently associated to worse functional outcomes.^22,23^

### Processing pipeline and features

The Arterial framework^19^ was used to compute geometrical and morphological features of the vascular segment spanning from the origin of the internal carotid artery (ICA) to the occlusion location. This segment was divided into two parts: first, the MCA segment, comprised between the ICA bifurcation extending up to the occlusion site. Second, the ICA segment, extending from the common carotid artery bifurcation to the ICA bifurcation, including both intra- and extracranial segments of the ICA. To that end, three landmark points were manually placed by one expert neurologist or one expert engineer on the baseline CTA for each patient: one at the common carotid artery bifurcation, one at the intracranial ICA bifurcation, and one at the arterial end immediately before the MDVO. Landmark annotations were performed using 3D Slicer.^24^ Manual annotation took <1 min per case.

Vascular centerlines for the individual segments were then automatically derived and characterized directly from the CTA using Arterial. Extracted features were based on centerline maps. For the MCA segment, these included the geodesic length (i.e. length along the centerline trajectory), the tortuosity index (TI; MCA-TI for the MCA segment), defined as:


(1)

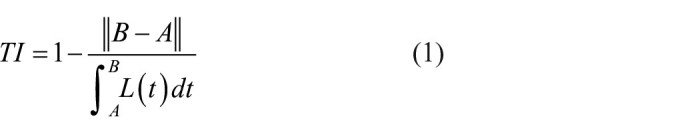



**Figure 2.**
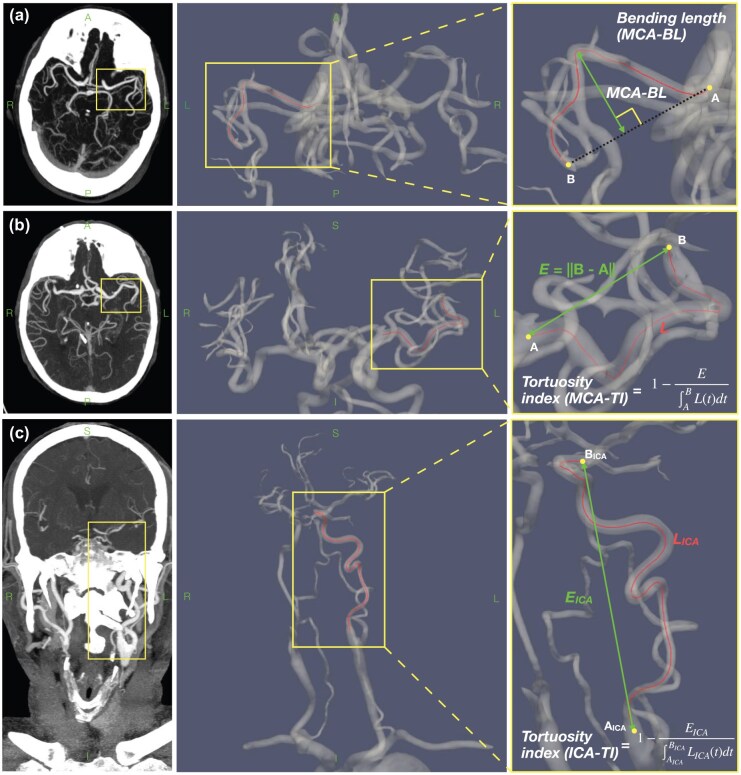
Illustration of the feature extraction methods for the (a) MCA-BL, (b) MCA-TI, and (c) ICA-TI. Left images show CTA view. Middle and right images show 3D view of the vascular segmentation automatically extracted by Arterial. On the right, an illustrative close-up feature definition can be found. BL: bending length; TI: tortuosity index; MCA: middle cerebral artery; ICA: internal carotid artery.

Where A is the proximal startpoint of the centerline, B is the distal endpoint, L(t) is the parametrized geodesic length, and ‖B−A‖ represents the Euclidean distance between A and B. The mean diameter along the MCA centerline segment (mean MCA diameter) and the mean diameter at the last 10 mm before the MDVO (diameter at occlusion) were also computed. The diameter measured with Arterial corresponds to the maximal inscribed sphere diameter at each centerline node. Finally, the bending length (MCA-BL)^25^ was also measured for the MCA segment, which corresponds to the maximal orthogonal distance between any point along the centerline and the imaginary axis defined between extremal points of the centerline (i.e. between A and B from equation (1)). For the ICA, only the TI (ICA-TI) was considered. ICA-TI was chosen as the sole descriptor of the ICA as it has been shown to be a strong predictor of reduced recanalization rates in multiple previous studies,^15,16,26^ it is quantifiable, and compared to other metrics based on angle measurements or curves, this feature is relatively free of ambiguity upon calculation. Figure 2 shows a schematic view of how MCA-BL, MCA-TI, and ICA-TI were extracted.

### Statistical analysis

Statistical difference between distributions across groups defined by binary endpoints (e.g. FPE vs no FPE) were measured by either the Student’s *t*-test, if the analyzed feature in both groups followed a normal distribution as described by the Shapiro-Wilk normality test, or the Mann-Whitney *U* test otherwise. The threshold for statistical significance in null hypothesis testing was set at *p* = 0.05. Results for distribution shift analysis are reported as the median with interquartile (IQR) values. Graphic representation of all feature distributions compared across study endpoints can be found in the Supplemental Material (section A.5). Unadjusted univariate logistic regression results were also computed and can be found in the Supplemental Material (section A.2).

The fundamental results of the study were assessed by logistic regression adjusted to time from symptoms onset to arterial puncture and administration of intravenous thrombolysis to explore the associations between anatomical features and the study endpoints. When fitting adjusted logistic regression to predict excellent functional outcomes, age and baseline mRS were included as additional adjustment variables. Adjusted odds ratio (aOR) with 95% confidence intervals (95%CI) were computed to describe the independent influence of each predictor over each endpoint. For TI features, values were multiplied by 10 in logistic regression analysis to improve interpretability of the aOR (i.e. the aOR value of the reflects the multiplication factor for the odds for an increment of 0.1 of the TI). Correlations between feature pairs were assessed by the Pearson correlation coefficient (*R*).

## Results

**Figure 3.**
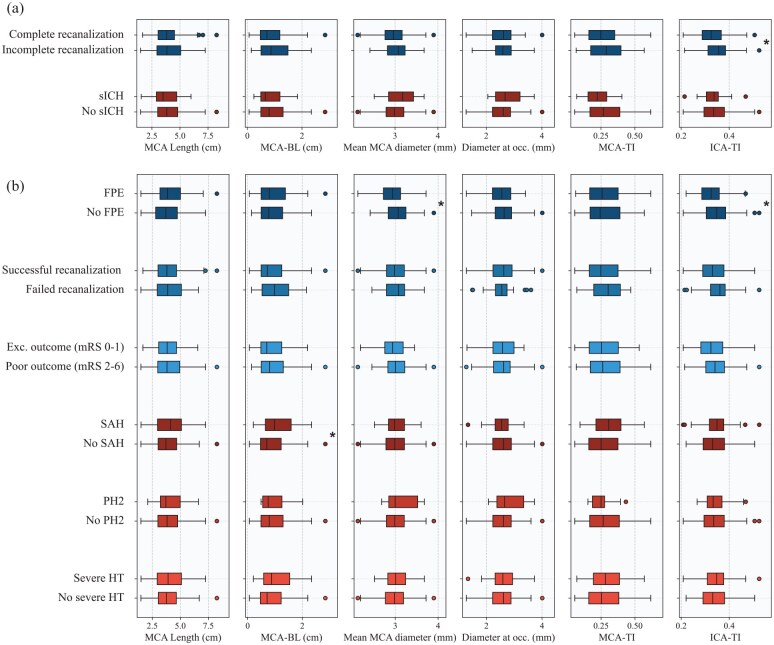
Distribution comparison of explored features across (a) primary and (b) secondary endpoints. Asterisks on the right side of the box plots indicate statistical significance as determined by either Student’s *t*-test or Mann-Whitney *U*-test, when appropriate. sICH: symptomatic intracranial hemorrhage; BL: bending length; MCA: middle cerebral artery; TI: tortuosity index; ICA: internal carotid artery; FPE: first pass effect; mRS: modified Rankin Scale; SAH: subarachnoid hemorrhage; PH2: parenchymal hematoma type 2; HT: hemorrhagic transformation.

**Figure 4.**
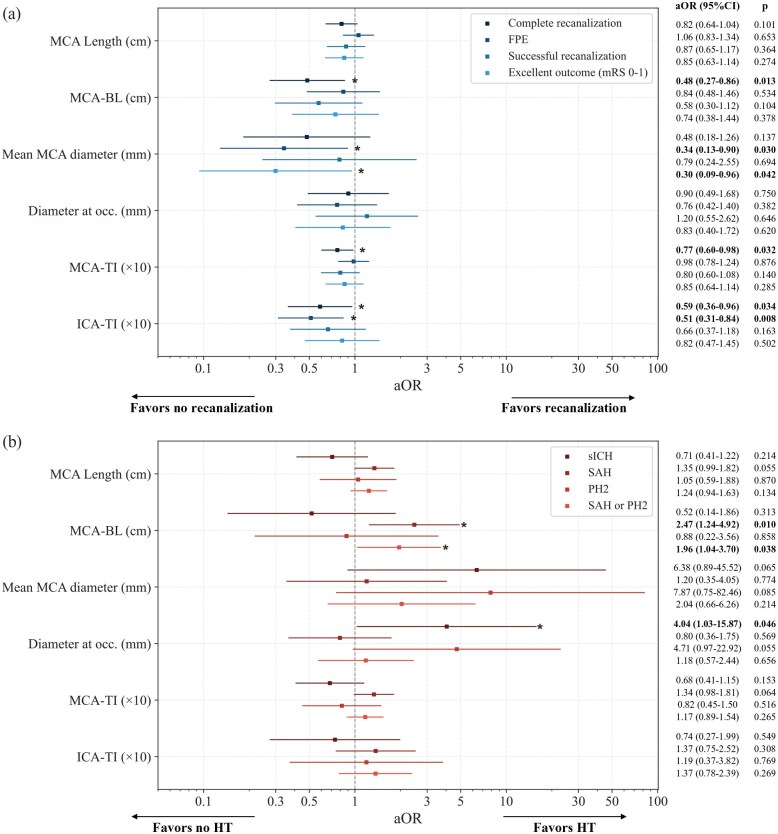
Results of the univariate logistic regression analysis (aOR with 95%CI) adjusted for time from symptoms onset to groin puncture and administration of intravenous thrombolysis for primary and secondary endpoints focusing on (a) treatment efficacy and (b) safety. Age and baseline mRS were also included as adjustment variables in the adjusted analysis for Excellent outcome (mRS 0–1). Numerical values for aOR and *p*-values for each feature-endpoint pair are displayed on the right. Asterisks and bold indicate statistical significance. sICH: symptomatic intracranial hemorrhage; BL: bending length; MCA: middle cerebral artery; TI: tortuosity index; ICA: internal carotid artery; FPE: first pass effect; mRS: modified Rankin Scale; aOR: adjusted odds ratio; SAH: subarachnoid hemorrhage; PH2: parenchymal hematoma type 2; HT: hemorrhagic transformation.

A total of 213 patients (81 years IQR 71–87, 51.2% women) were included in the final sample. The most frequent location was the M2 segment (proximal M2: 100, 46.9%; distal M2: 103, 48.4%), while the remaining cases were M3 occlusions (10, 4.7%). Baseline characteristics, procedural and clinical outcomes and feature measurements for the whole population and for groups segregated by the primary endpoints can be found in Table 1. An equivalent table with groups defined by secondary endpoints can be found in the Supplemental Material (Table A1). Figure 3 shows a comparison of measured feature distributions across dichotomized groups for all endpoints. Results for the unadjusted logistic regression analysis can be found in the Supplemental Material (Figure A1). Results for the logistic regression analysis can be found in Figure 4.

**Table 1.** Baseline characteristics, procedural and clinical outcomes for the full sample, as well as for groups defined by the primary endpoints.

Characteristic	Whole sample	Complete recanalization (eTICI 2 c/3)		sICH	
	*n* = 213	Yes (129, 59.7%)	No (87, 40.3%)	No (202, 93.3%)	Yes (14, 6.7%)
Age	81 (72–87)	82 (73–87)	80 (71–86)	81 (72–87)	81 (74–87)
Sex	109 (51.2%)	66 (51.2%)	43 (51.2%)	104 (52.3%)	5 (35.7%)
Left-sided	125 (58.7%)	75 (58.1%)	50 (59.5%)	118 (59.3%)	7 (50.0%)
NIHSS on admission	11 (7–17)	11 (7–17)	11 (8–16)	**11 (8–17)**	**6 (5–10)**
Baseline mRS	1 (0–2)	1 (0–2)	1 (0–2)	1 (0–2)	2 (1–3)
IVT	62 (29.1%)	36 (27.9%)	26 (31.0%)	57 (28.6%)	5 (35.7%)
Occluded vessel					
Proximal M2	100 (46.9%)	62 (48.1%)	38 (45.2%)	93 (46.7%)	7 (50.0%)
Distal M2	103 (48.4%)	60 (46.5%)	43 (51.2%)	96 (48.2%)	7 (50.0%)
M3	10 (4.7%)	7 (5.4%)	3 (3.6%)	10 (5.0%)	0 (0.0%)
eTICI (first pass)					
0	66 (31.0%)	***24 (18.6%)***	***42 (50.0%)***	60 (30.2%)	6 (42.9%)
1	6 (2.8%)	*2 (1.6%)*	*4 (4.8%)*	5 (2.5%)	1 (7.1%)
2a	19 (8.9%)	***7 (5.4%)***	***12 (14.3%)***	17 (8.5%)	2 (14.3%)
2b	29 (13.6%)	***4 (3.1%)***	***25 (29.8%)***	27 (13.6%)	2 (14.3%)
2c	35 (16.4%)	***34 (26.4%)***	***1 (1.2%)***	35 (17.6%)	0 (0.0%)
3	58 (27.2%)	***58 (45.0%)***	***0 (0.0%)***	55 (27.6%)	3 (21.4%)
Final eTICI					
0	30 (14.1%)	***0 (0.0%)***	***30 (35.7%)***	**25 (12.6%)**	**5 (35.7%)**
1	1 (0.5%)	*0 (0.0%)*	*1 (1.2%)*	1 (0.5%)	0 (0.0%)
2a	9 (4.2%)	***0 (0.0%)***	***9 (10.7%)***	9 (4.5%)	0 (0.0%)
2b	44 (20.7%)	***0 (0.0%)***	***44 (52.4%)***	40 (20.1%)	4 (28.6%)
2c	56 (26.3%)	***56 (43.4%)***	***0 (0.0%)***	54 (27.1%)	2 (14.3%)
3	73 (34.3%)	***73 (56.6%)***	***0 (0.0%)***	70 (35.2%)	3 (21.4%)
Number of passes	1 (1–2)	***1 (1–2)***	***2 (1–3)***	1 (1–2)	1 (1–2)
Hemorrhagic events					
sICH	14 (6.6%)	5 (3.9%)	9 (10.7%)	***0 (0.0%)***	***14 (100.0%)***
SAH	38 (17.8%)	**17 (13.2%)**	**21 (25.0%)**	***31 (15.6%)***	***7 (50.0%)***
PH2	10 (4.7%)	3 (2.3%)	7 (8.3%)	***2 (1.0%)***	***8 (57.1%)***
Severe HT (PH2 or SAH)	47 (22.1%)	**19 (14.7%)**	**28 (33.3%)**	***33 (16.6%)***	***14 (100.0%)***
NIHSS at 24 h	7 (3–15)	**4 (1–11)**	**11 (6–18)**	**7 (3–13)**	**18 (13–29)**
NIHSS at discharge	3 (1–8)	**2 (1–5)**	**7 (3–11)**	**3 (1–7)**	**13 (8–17)**
mRS at 90 days	3 (1–4)	**2 (1–4)**	**3 (2–5)**	**3 (1–4)**	**5 (4–6)**
MCA Length (cm)	3.82 (3.02–4.78)	3.80 (3.06–4.51)	3.84 (2.95–5.06)	3.83 (3.02–4.80)	3.49 (2.90–4.71)
MCA-BL (cm)	0.79 (0.49–1.31)	0.72 (0.47–1.19)	0.87 (0.50–1.49)	0.80 (0.50–1.31)	0.66 (0.49–1.19)
Mean MCA diameter (mm)	2.99 (2.80–3.22)	2.96 (2.77–3.16)	3.08 (2.82–3.23)	2.98 (2.79–3.20)	3.18 (2.85–3.43)
Diameter at occlusion (mm)	2.61 (2.24–2.90)	2.63 (2.22–2.89)	2.60 (2.34–2.90)	2.61 (2.23–2.87)	2.68 (2.34–3.21)
MCA-TI	0.26 (0.17–0.38)	0.25 (0.16–0.35)	0.29 (0.17–0.40)	0.27 (0.17–0.39)	0.22 (0.15–0.29)
ICA-TI	0.34 (0.30–0.38)	**0.33 (0.29–0.37)**	**0.36 (0.31–0.38)**	0.34 (0.30–0.38)	0.34 (0.31–0.35)

sICH: symptomatic intracranial hemorrhage; BL: bending length; MCA: middle cerebral artery; TI: tortuosity index; ICA: internal carotid artery; FPE: first pass effect; mRS: modified Rankin Scale; SAH: subarachnoid hemorrhage; PH2: parenchymal hematoma type 2; HT: hemorrhagic transformation; NIHSS: National Institute of Health Stroke Scale; IVT: intravenous thrombolysis; eTICI: expanded thrombolysis in cerebral infarction.

Results for numerical variables are displayed with the median and IQR values. Distributions that present statistical differences across groups (i.e. *p* < 0.05 in χ^2^, Student’s *t-*test or Mann-Whitney *U*-test when appropriate) are highlighted in bold. Italics indicate characteristics that are by definition highly correlated or equivalent to the grouping variable.

### Primary endpoints

Complete recanalization (final eTICI 2c/3) was achieved in 59.7% (129/213) of patients. In unadjusted feature distribution analysis, only ICA-TI was significantly different in patients where complete recanalization (0.33 IQR [0.29–0.37] vs 0.36 [0.31–0.38], *p* = 0.007) was not achieved. However, in addition to ICA-TI (aOR 0.59 [95% CI 0.36–0.96], *p* = 0.034), MCA tortuosity was also associated with lower odds of complete recanalization in adjusted logistic regression analysis, both in terms of the MCA-BL (aOR 0.48 [0.27–0.86], *p* = 0.013) and MCA-TI (aOR 0.77 [0.60–0.98], *p* = 0.032).

Regarding sICH (6.7%, 14/213), the primary safety endpoint, larger diameter at the occlusion site was significantly associated with sICH (aOR 4.04 [1.03–15.87], *p* = 0.046) in adjusted logistic regression. No significant differences were found across distributions in any of the explored descriptors on unadjusted analysis.

### Secondary endpoints

Regarding secondary efficacy endpoints, patients achieving FPE (93/213, 43.7%) presented smaller mean MCA diameters (2.94 [2.72–3.12] mm vs 3.07 [2.84–3.25] mm, *p* = 0.005) and lower ICA-TI (0.33 [0.29–0.36] vs 0.35 [0.30–0.38], *p* = 0.008). These results remained significant in the adjusted logistic regression analyses (mean MCA diameter w/ FPE: aOR 0.34 [0.13–0.90], *p* = 0.030; ICA-TI w/ FPE: aOR 0.51 [0.31–0.84], *p* = 0.008). No significant influence of vascular anatomic features was observed on successful recanalization rates (173/213, 81.2%).

Excellent outcome was observed in 61/213 (28.6%) of patients. Patients with larger mean MCA diameter were less likely to achieve an excellent functional outcome at 3 months (aOR 0.30 [0.09–0.96], *p* = 0.042).

Regarding secondary safety endpoints, SAH was observed in 38/213 patients (17.8%), PH2 in 10/213 patients (4.7%) and the severe HT composite in 47/213 patients (22.1%). The MCA-BL was significantly higher in patients with SAH (0.70 [0.47–1.23] vs 0.98 [0.65–1.58], *p* = 0.023). In adjusted analysis, higher MCA-BL was significantly associated with SAH (aOR 2.47 [1.24–4.92], *p* = 0.010) and severe HT (aOR 1.96 [1.04–3.70], *p* = 0.038). No features showed a statistically significant correlation with PH2; however, there was a trend suggesting that larger diameters may be associated with increased likelihood of PH2 (Figure 4). ICA-TI did not show correlation with any safety secondary endpoints.

### Correlation across features

Correlation plots can be found in the Supplemental Material (section A.4, Figure A3). MCA-BL was positively correlated with MCA length (*R* = 0.914, *p* < 0.001) and MCA-TI (0.874, *p* < 0.001), and negatively correlated with the diameter at occlusion (−0.468, *p* < 0.001) and mean MCA diameter (−0.397, *p* < 0.001). Mean MCA diameter was strongly correlated with diameter at occlusion (0.853, *p* < 0.001).

No correlation was observed between ICA-TI and cerebral tortuosity (ICA-TI vs MCA-BL: −0.069, *p* = 0.316; ICA-TI vs MCA-TI: 0.003, *p* = 0.971), or between ICA-TI and cerebral diameter (ICA-TI vs mean MCA diameter: 0.088, *p* = 0.201; ICA-TI vs diameter at occlusion: −0.022, *p* = 0.760).

## Discussion

This exploratory study deepens into understanding, from a quantitative standpoint, how intracranial vascular tortuosity may influence outcomes of MT for MDVO with currently available thrombectomy devices. Neutral results from recent trials testing the efficacy of MT in patients with MDVO^8,9^ underscore the need for more precise triage strategies in this population, in order to better identify those patients in which MT will be most safe and effective. Vascular anatomy may play an important role in this context.^7,14^

Previous studies have shown that anatomic vascular features may influence MT outcomes.^12,16,17,26,27^ However, the lack of a rapid, standardized assessment precluded the adoption of such criteria in prospective studies. In our study, we used a proprietary AI-based framework that allowed consistent rapid semi-automated independent extraction of multiple anatomic vascular features from the baseline CTA file. If the extracted features confirm their value in optimizing patient selection, future developments to design a fully automated platform capable of offering a detailed anatomic vascular characterization in real time should be considered.

The framework showed that ICA-TI had a strong influence over MT recanalization outcomes. However, the odds of hemorrhagic events were not influenced by ICA tortuosity. Conversely, cerebral tortuosity, particularly the MCA-BL, was linked to both a lower rate of complete recanalization and increased odds of SAH and severe HT (i.e. SAH and/or PH2). The MCA-BL of the studied MCA segment can be interpreted as a quantitative approximation that describes a combination of the MCA length and elongation. At such distal territories, control over proper device positioning as well as force transmission efficiency in the clot-device interaction upon retrieval^13,27^ may be affected by friction with arterial walls, more substantially than in proximal LVO thrombectomies. These interferences may be enhanced by a pronounced MCA elongation and intracranial tortuosity, introducing excessive stress over vessel walls and ultimately leading to inefficient thrombus retrieval, propensity to vessel collapse, displacement and deformation^28^ or even higher risk of vessel damage and avulsion.^29^

A larger mean MCA diameter was associated with lower probability of FPE, in line with previous literature,^18^ and lower likelihood of excellent functional outcomes. Previous review studies have reported improved recanalization rates with the combined technique (stent retriever + aspiration) as firstline MT strategy over single-device setups^30^ in MDVOs treatment. In our study cohort, the combined technique was the most frequently used (combined: 90.4%; stent-retriever: 2.3%; aspiration 7.1%). Higher diameter ratios of distal access catheter (DAC) to artery lumen (i.e. catheter covering most of the internal vessel diameter) have been linked to improved recanalization rates, an effect that is attributed to a facilitated ingestion of the clot into the catheter and to the flow arrest caused by larger devices.^31^ The navigation of larger DACs, easily delivered to proximal occlusion sites, may be challenging through tortuosities in cases of distal occlusions making it more difficult to achieve the same catheter to vessel diameter ratio. Therefore, in MDVO a larger diameter at the site of occlusion may have a stronger negative impact than in proximal LVOs, decreasing the efficacy of the procedure in terms of recanalization (Figures 3 and 4). Future developments in device engineering may allow an improved and safe navigation of larger devices into distal vessels, optimizing the catheter to vessel diameter ratio and the recanalization outcomes in MDVO.

The negative effect of larger mean MCA diameter on functional outcomes may be more challenging to interpret, as there are many potential co-factors that were not considered in the study. We hypothesize that effects derived from inefficient thrombus retrieval in the first MT pass, such as a higher risk of distal embolization due to decreased efficiency of smaller 3 mm SR when retrieved through larger proximal arteries, or vessel damage from subsequent passes, may lead to worse long-term outcomes. Potential derivations from an inefficient first pass could also explain the association of larger diameter at the occlusion with increased risk of sICH. However, these results should be interpreted with caution and ideally validated with a larger and more heterogeneous sample, as the small number of cases with sICH in the present population (14/213, 6.7%) was rather low.

Measurements for diameter at occlusion (2.57 ± 0.47 mm) were slightly larger than reported in previous literature (estimated around 2.4 mm at proximal M2).^7,18^ Segmentation-based measurements are constrained by the native image voxel size, which was approximately 0.4 mm in all directions in our case. As a result, we can expect an error distribution greater or equal than this magnitude, which is comparable to the vessel size in distal territories. Qualitatively, we observed a slight tendency of the model to over-segment small vessels. This could result in a systemic bias upward of the used measurement methodology, primarily affecting nominal diameter estimates, while having a more limited impact on the core findings of the study, such as statistical associations and group differences.

The vascular anatomy description of the studied segment was reduced to a small number of features; measurements such as angles between consecutive segments or presence of coiling or kinking were not considered, which may have an impact over treatment efficacy and safety as well.^12,27^ The primary motivation behind selecting this feature set was to maintain a strong descriptive power of the intracranial arterial anatomy relevant in the MT procedure, while avoiding ill-defined descriptors that currently lack consensus in terms of definition and acquisition methodology. Instead, we focused on features that are well-defined and more readily compatible with our existing system, also considering practical implementation in a hypothetical future software deployment, facilitating reproducibility, interpretability and clarity.

Moreover, while most features describing cerebral vascular anatomy were highly inter-correlated between one another, no correlation was observed between tortuosity descriptors of the MCA segment and ICA-TI. Since markers from both anatomical segments were observed to be predictive of poor thrombectomy outcomes, the lack of correlation hints that the combination of both may offer superior discrimination performance than either on its own. This suggests that future predictive models should try to incorporate information from both segments to be as performant as possible. The characterization of the extracranial arterial anatomy may also have a considerable impact over studied endpoints in this particular population with MDVOs, but this research question falls out of the scope of the main hypothesis of the study.

Fast, automated and reproducible characterization methods of the arterial anatomy, together with data-driven predictive models, may enable advanced triage tools in the near future, allowing practitioners to accurately select patients that are likely to benefit from treatment and present low risk of safety complications, ultimately identifying patients in which MT might be superior to best medical treatment. Ideally, these models should incorporate additional information on top of vascular anatomical descriptors such as radiomic and morphologic characteristics of the clot or clinical variables.

One limitation of our approach is that we could not include characteristics of the occluded arterial segments. Vascular morphology along the occlusion, particularly in occlusions at bifurcations, may have a strong impact on recanalization effectiveness.^32^ However, these segments were not visible on CTA due to radiological homogeneity of clot material with surrounding brain tissue or poor contrast arrival, making their analysis challenging.

Other limitations include the retrospective nature of the study and the inclusion of patients from a single center. Additionally, we did not include patients with anterior cerebral artery or posterior cerebral artery MDVOs, which were included in recent trials,^8,9^ and we did not make a distinction according to M2 branch dominance. Our cohort included predominantly patients treated with the combined technique, and therefore comparisons of the impact of anatomical features on different devices was not possible. It is likely that tortuosities may affect each device type differently. Further studies are needed to determine if such analyses could also help neurointerventionalists individualizing device selection based on the patients’ anatomical features.

Future work will focus on validating these findings with patients from external and larger cohorts, including patients with a wider variety of MDVO territories, as well as working toward building predictive models inferring treatment efficacy and safety risks based on anatomical information and thrombus radiological characteristics.

## Conclusions

In patients with MDVO in the MCA M2/M3 territories who received mechanical thrombectomy, intracranial vascular tortuosity was associated with lower odds of complete recanalization and increased risk of hemorrhagic transformation. The MCA bending length emerged as a strong predictor for both MT efficacy and safety complications. The tortuosity of the ICA was associated with lower odds of recanalization efficacy, but not with safety endpoints. Larger mean MCA diameter was correlated with lower odds of first pass recanalization and worse functional outcome, while larger diameter at the occlusion site was associated with increased risk of sICH. Quantitative anatomical measurements could be used to define objective and effective triage criteria in the future.

## Supplementary Material

sj-pdf-1-eso-23969873251350124

## Data Availability

Data used in this study cannot be publicly shared but can be made available upon reasonable request.
