# Supplementary material for: Prognostic value of intracranial vascular tortuosity in thrombectomy for distal vessel occlusion
Source: Eur Stroke J. 2026 Jan 1;11(1):23969873251350124. doi: 10.1093/esj/23969873251350124 (PMC12866268; doi:10.1093/esj/23969873251350124)
Supplement: sj-pdf-1-eso-23969873251350124 [file sj-pdf-1-eso-23969873251350124.pdf]

Supplementary material

Prognostic Value of Intracranial Vascular Tortuosity in Thrombectomy for Distal Vessel Occlusion

Appendix A - Additional analyses

A.1 Baseline characteristics and features for secondary endpoints

Baseline characteristics, procedural and clinical outcomes as well as feature measurements for groups defined by secondary endpoints can be found in table A1.

	FPE (eTICI 2c/3 @ first pass)		Successful recanalization (eTICI 2b/3)		Excellent outcome (mRS 0-1)		SAH		PH2		Severe HT (PH2 or SAH)	
	Yes (93, 43.7%)	No (120, 43.7%)	Yes (173, 81.2%)	No (40, 18.8%)	Yes (61, 28.6%)	No (152, 71.4%)	No (175, 82.2%)	Yes (38, 17.8%)	No (203, 95.3%)	Yes (10, 4.7%)	No (166, 77.9%)	Yes (47, 22.1%)
**Age**	82 (74-87)	81 (71-87)	81 (71-87)	81 (73-87)	**75 (66-82)**	**84 (75-88)**	81 (71-87)	82 (73-87)	82 (72-87)	80 (65-80)	81 (71-86)	81 (74-87)
**Sex**	48 (51.6%)	61 (50.8%)	87 (50.3%)	22 (55.0%)	26 (42.6%)	83 (54.6%)	91 (52.0%)	18 (47.4%)	103 (50.7%)	6 (60.0%)	86 (51.8%)	23 (48.9%)
**Left-sided**	52 (55.9%)	73 (60.8%)	99 (57.2%)	26 (65.0%)	31 (50.8%)	94 (61.8%)	104 (59.4%)	21 (55.3%)	121 (59.6%)	4 (40.0%)	99 (59.6%)	26 (55.3%)
**NIHSS on admission**	10 (7-17)	11 (8-16)	10 (7-16)	13 (8-17)	**9 (7-14)**	**11 (8-17)**	10 (7-17)	12 (8-15)	11 (8-16)	9 (5-15)	10 (7-16)	12 (8-16)
**Baseline mRS**	1 (0-2)	1 (0-2)	1 (1-2)	1 (0-2)	**1 (0-1)**	**2 (1-2)**	1 (0-2)	1 (1-2)	1 (0-2)	2 (0-3)	1 (0-2)	1 (0-2)
**IVT**	25 (26.9%)	37 (30.8%)	48 (27.7%)	14 (35.0%)	19 (31.1%)	43 (28.3%)	53 (30.3%)	9 (23.7%)	59 (29.1%)	3 (30.0%)	50 (30.1%)	12 (25.5%)
**Occluded vessel**												
M2 (proximal)	40 (43.0%)	60 (50.0%)	83 (48.0%)	17 (42.5%)	31 (50.8%)	69 (45.4%)	87 (49.7%)	13 (34.2%)	96 (47.3%)	4 (40.0%)	82 (49.4%)	18 (38.3%)
M2 (distal)	46 (49.5%)	57 (47.5%)	83 (48.0%)	20 (50.0%)	27 (44.3%)	76 (50.0%)	79 (45.1%)	24 (63.2%)	97 (47.8%)	6 (60.0%)	75 (45.2%)	28 (59.6%)
M3	7 (7.5%)	3 (2.5%)	7 (4.0%)	3 (7.5%)	3 (4.9%)	7 (4.6%)	9 (5.1%)	1 (2.6%)	10 (4.9%)	0 (0.0%)	9 (5.4%)	1 (2.1%)
**eTICI (first pass)**												
0	***0 (0.0%)***	***66 (55.0%)***	***34 (19.7%)***	***32 (80.0%)***	**10 (16.4%)**	**56 (36.8%)**	**48 (27.4%)**	**18 (47.4%)**	61 (30.0%)	5 (50.0%)	**43 (25.9%)**	**23 (48.9%)**
1	*0 (0.0%)*	*6 (5.0%)*	*3 (1.7%)*	*3 (7.5%)*	1 (1.6%)	5 (3.3%)	**2 (1.1%)**	**4 (10.5%)**	6 (3.0%)	0 (0.0%)	2 (1.2%)	4 (8.5%)
2a	***0 (0.0%)***	***19 (15.8%)***	*15 (8.7%)*	*4 (10.0%)*	4 (6.6%)	15 (9.9%)	17 (9.7%)	2 (5.3%)	18 (8.9%)	1 (10.0%)	16 (9.6%)	3 (6.4%)
2b	***0 (0.0%)***	***29 (24.2%)***	***29 (16.8%)***	***0 (0.0%)***	8 (13.1%)	21 (13.8%)	22 (12.6%)	7 (18.4%)	28 (13.8%)	1 (10.0%)	21 (12.7%)	8 (17.0%)
2c	***35 (37.6%)***	***0 (0.0%)***	***34 (19.7%)***	***1 (2.5%)***	**16 (26.2%)**	**19 (12.5%)**	32 (18.3%)	3 (7.9%)	35 (17.2%)	0 (0.0%)	32 (19.3%)	3 (6.4%)
3	***58 (62.4%)***	***0 (0.0%)***	***58 (33.5%)***	***0 (0.0%)***	22 (36.1%)	36 (23.7%)	**54 (30.9%)**	**4 (10.5%)**	55 (27.1%)	3 (30.0%)	**52 (31.3%)**	**6 (12.8%)**
**Final eTICI**												
0	***1 (1.1%)***	***29 (24.2%)***	***0 (0.0%)***	***30 (75.0%)***	**1 (1.6%)**	**29 (19.1%)**	21 (12.0%)	9 (23.7%)	**25 (12.3%)**	**5 (50.0%)**	**16 (9.6%)**	**14 (29.8%)**
1	*0 (0.0%)*	*1 (0.8%)*	*0 (0.0%)*	*1 (2.5%)*	0 (0.0%)	1 (0.7%)	0 (0.0%)	1 (2.6%)	1 (0.5%)	0 (0.0%)	0 (0.0%)	1 (2.1%)
2a	***0 (0.0%)***	***9 (7.5%)***	*0 (0.0%)*	*9 (22.5%)*	1 (1.6%)	8 (5.3%)	6 (3.4%)	3 (7.9%)	9 (4.4%)	0 (0.0%)	6 (3.6%)	3 (6.4%)
2b	***0 (0.0%)***	***44 (36.7%)***	***44 (25.4%)***	***0 (0.0%)***	11 (18.0%)	33 (21.7%)	36 (20.6%)	8 (21.1%)	42 (20.7%)	2 (20.0%)	34 (20.5%)	10 (21.3%)
2c	***33 (35.5%)***	***23 (19.2%)***	***56 (32.4%)***	***0 (0.0%)***	**24 (39.3%)**	**32 (21.1%)**	48 (27.4%)	8 (21.1%)	56 (27.6%)	0 (0.0%)	48 (28.9%)	8 (17.0%)
3	***59 (63.4%)***	***14 (11.7%)***	***73 (42.2%)***	***0 (0.0%)***	24 (39.3%)	49 (32.2%)	64 (36.6%)	9 (23.7%)	70 (34.5%)	3 (30.0%)	62 (37.3%)	11 (23.4%)
**Number of passes**	***1 (1-1)***	***2 (1-3)***	**1 (1-2)**	**3 (2-4)**	**1 (1-2)**	**1 (1-3)**	**1 (1-2)**	**2 (1-3)**	1 (1-2)	1 (1-3)	**1 (1-2)**	**2 (1-3)**
**Hemorrhagic events**												
sICH	3 (3.2%)	11 (9.2%)	9 (5.2%)	5 (12.5%)	**0 (0.0%)**	**14 (9.2%)**	*7 (4.0%)*	*7 (18.4%)*	***6 (3.0%)***	***8 (80.0%)***	***0 (0.0%)***	***14 (29.8%)***
SAH	**7 (7.5%)**	**31 (25.8%)**	**25 (14.5%)**	**13 (32.5%)**	7 (11.5%)	31 (20.4%)	***0 (0.0%)***	***38 (100.0%)***	36 (17.7%)	2 (20.0%)	***0 (0.0%)***	***38 (80.9%)***
PH2	**3 (3.2%)**	**7 (5.8%)**	**5 (2.9%)**	**5 (12.5%)**	0 (0.0%)	10 (6.6%)	8 (4.6%)	2 (5.3%)	***0 (0.0%)***	***10 (100.0%)***	***0 (0.0%)***	***10 (21.3%)***
HT (PH2/SAH/sICH)	**9 (9.7%)**	**38 (31.7%)**	**29 (16.8%)**	**18 (45.0%)**	**7 (11.5%)**	**40 (26.3%)**	***9 (5.1%)***	***38 (100.0%)***	***37 (18.2%)***	***10 (100.0%)***	***0 (0.0%)***	***47 (100.0%)***
**NIHSS at 24h**	**3 (1-8)**	**10 (6-18)**	**6 (2-12)**	**14 (8-18)**	**2 (0-5)**	**11 (5-18)**	**6 (2-13)**	**11 (6-18)**	**7 (3-13)**	**18 (18-25)**	**6 (2-12)**	**13 (8-18)**
**NIHSS at discharge**	**2 (0-4)**	**5 (2-9)**	**3 (1-6)**	**9 (5-17)**	**1 (0-2)**	**6 (3-11)**	**3 (1-7)**	**7 (4-12)**	**3 (1-7)**	**13 (8-17)**	**3 (1-6)**	**7 (4-16)**
**mRS at 90d**	**2 (1-4)**	**3 (2-5)**	**3 (1-4)**	**3 (2-6)**	***1 (0-1)***	***3 (3-6)***	**3 (1-4)**	**3 (2-6)**	**3 (1-4)**	**4 (4-5)**	**3 (1-4)**	**4 (2-6)**
**MCA Length (cm)**	3.87 (3.19-5.01)	3.72 (2.81-4.74)	3.80 (3.02-4.66)	3.88 (2.95-5.10)	3.83 (3.07-4.66)	3.81 (2.99-4.95)	3.72 (3.02-4.67)	4.13 (2.97-5.10)	3.82 (3.01-4.76)	3.71 (3.23-4.99)	3.76 (3.04-4.65)	3.91 (2.94-5.10)
**MCA-BL (cm)**	0.80 (0.49-1.38)	0.77 (0.50-1.28)	0.72 (0.49-1.25)	0.98 (0.53-1.50)	0.70 (0.49-1.25)	0.81 (0.50-1.31)	**0.70 (0.47-1.23)**	**0.98 (0.65-1.59)**	0.79 (0.49-1.30)	0.75 (0.55-1.26)	0.71 (0.47-1.23)	0.88 (0.59-1.54)
**Mean MCA diameter (mm)**	**2.94 (2.72-3.12)**	**3.07 (2.84-3.25)**	2.98 (2.80-3.21)	3.08 (2.79-3.22)	2.94 (2.76-3.18)	3.01 (2.83-3.22)	2.99 (2.78-3.21)	2.98 (2.84-3.22)	2.99 (2.79-3.21)	3.00 (2.85-3.52)	2.98 (2.77-3.19)	3.01 (2.83-3.24)
**Diameter at occlusion (mm)**	2.56 (2.22-2.88)	2.64 (2.32-2.91)	2.63 (2.24-2.92)	2.56 (2.33-2.74)	2.58 (2.24-2.99)	2.62 (2.26-2.85)	2.62 (2.23-2.90)	2.55 (2.31-2.78)	2.61 (2.23-2.89)	2.65 (2.38-3.34)	2.62 (2.23-2.88)	2.59 (2.31-2.94)
**MCA-TI**	0.26 (0.17-0.38)	0.24 (0.17-0.39)	0.25 (0.16-0.37)	0.30 (0.20-0.39)	0.25 (0.16-0.38)	0.26 (0.17-0.39)	0.25 (0.16-0.37)	0.31 (0.21-0.40)	0.27 (0.17-0.38)	0.25 (0.19-0.28)	0.25 (0.16-0.38)	0.28 (0.19-0.39)
**ICA-TI**	**0.33 (0.29-0.36)**	**0.35 (0.30-0.38)**	0.33 (0.29-0.38)	0.36 (0.32-0.38)	0.32 (0.28-0.37)	0.34 (0.30-0.38)	0.33 (0.29-0.38)	0.35 (0.32-0.38)	0.34 (0.30-0.38)	0.33 (0.31-0.37)	0.33 (0.29-0.38)	0.35 (0.31-0.38)

Table A1. Baseline characteristics, procedural and clinical outcomes for groups defined by the secondary endpoints. Results for nur variables are displayed with the median and IQR values. Distributions that present statistical differences across groups (i.e., p<0.05 Student's t-test or Mann-Whitney U-test when appropriate) are highlighted in bold. sICH: symptomatic intracranial hemorrhage. BL: b length. MCA: middle cerebral artery. TI: tortuosity index. ICA: internal carotid artery. FPE: first pass effect. mRS: modified Rankin SAH: subarachnoid hemorrhage. PH2: parenchymal hematoma type 2. HT: hemorrhagic transformation. NIHSS: National Institute of Stroke Scale. IVT: intravenous thrombolysis. eTICI: expanded thrombolysis in cerebral infarction.

A.2 Unadjusted logistic regression analysis

Results for the unadjusted logistic regression analysis can be found in this section. Common odds ratios (cOR) are reported for univariate regression analysis in figure A1.

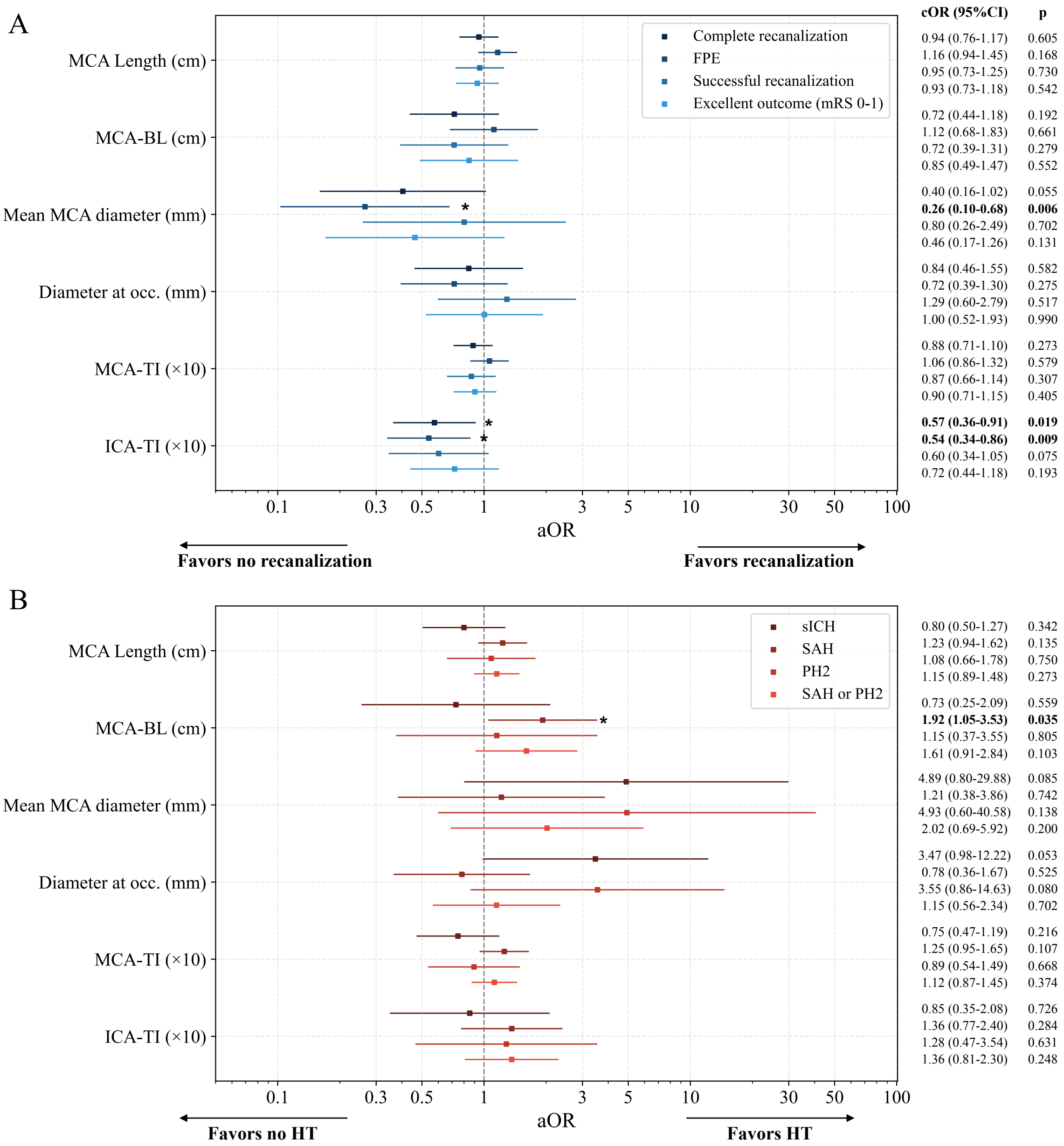

Figure A1. Results of the univariate logistic regression analysis (cOR with 95%CI) for primary and secondary endpoints focusing on (A) treatment efficacy and (B) safety. Numerical values for cOR and p-values for each feature-endpoint pair are displayed on the right. Asterisks and bold indicate statistical significance. sICH: symptomatic intracranial hemorrhage. BL: bending length. MCA: middle cerebral artery. TI: tortuosity index. ICA: internal carotid artery. FPE: first pass effect. mRS: modified Rankin Scale. cOR: common odds ratio. SAH: subarachnoid hemorrhage. PH2: parenchymal hematoma type 2. HT: hemorrhagic transformation.

Upon unadjusted analysis, no associations were found between tortuosity features of the cerebral segments and efficacy endpoints, except for the mean diameter, which was associated with a decreased chance of first-pass recanalization (cOR 0.26, [95% CI 0.10-0.68], p=0.006). High ICA-TI were associated with lower rates of complete recanalization (cOR 0.57 [0.36-0.91], p=0.019) and first-pass recanalization (cOR 0.54 [0.34-0.86], p=0.009). The BL was the only feature that was significantly associated with any of the safety endpoints (SAH, cOR 1.92 [1.05-3.35], p=0.035) on unadjusted analysis.

A.3 Adjustment variables association to endpoints

In this section, results for adjustment variables employed in the adjusted analysis are displayed for all studied endpoints. These can be seen in figure A2.

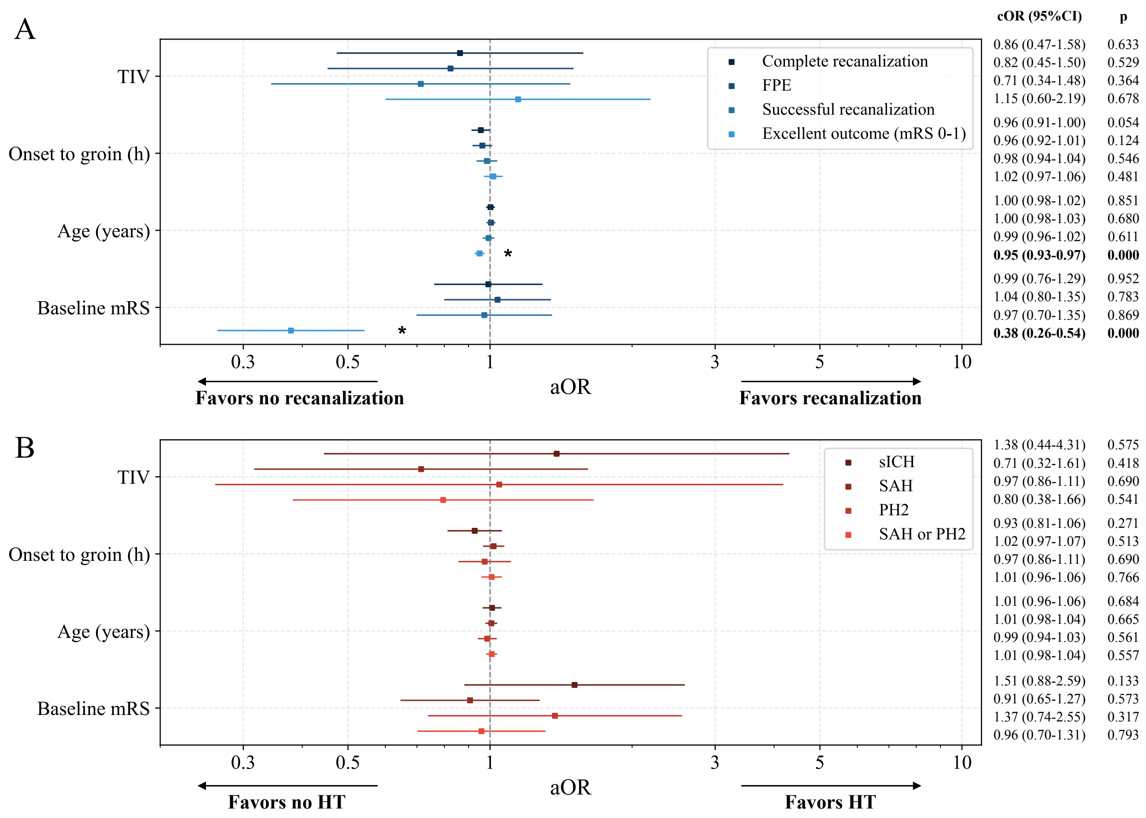

Figure A2. Results of the univariate logistic regression analysis (cOR with 95%CI) of adjustment variables for primary and secondary endpoints focusing on (A) treatment efficacy and (B) safety. Numerical values for cOR and p-values for each feature-endpoint pair are displayed on the right. Asterisks and bold indicate statistical significance. sICH: symptomatic intracranial hemorrhage. FPE: first pass effect. mRS: modified Rankin Scale. cOR: common odds ratio. SAH: subarachnoid hemorrhage. PH2: parenchymal hematoma type 2. HT: hemorrhagic transformation.

Figure A2 shows how that baseline mRS and age were both significantly associated with Excellent outcome (mRS 0-1) at three months. No other significant associations were found across the rest of potential descriptors and study endpoints. However, based these were added to adjusted logistic regression analysis based on clinical plausibility and previous evidence available in the literature

A.4 Correlation plots between features

Correlation plots across feature pairs included in the study can be found in figure A3.

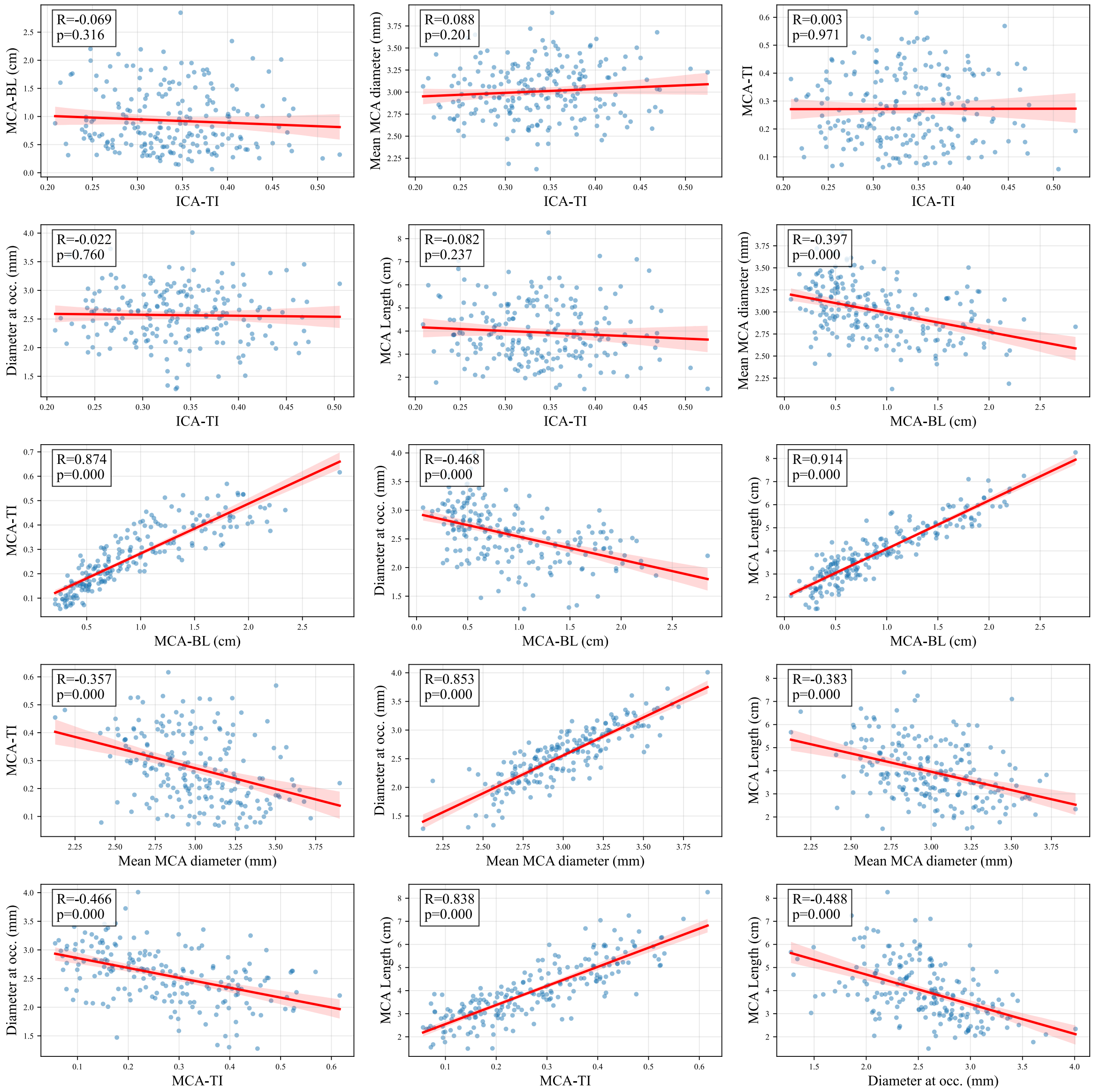

Figure A3. Correlation plots across analyzed feature pairs. Pearson's correlation coefficient (R) and p-value are displayed. BL: bending length. MCA: middle cerebral artery. TI: tortuosity index. ICA: internal carotid artery.

A.5 Feature distributions across endpoints

Feature distributions across endpoints are displayed in this section as histograms with normalized Kernel density estimation (KDE) plots. Distributions per groups defined by primary endpoints can be found in figure A4, while distributions per secondary efficacy and safety are shown in figures A5 and A6.

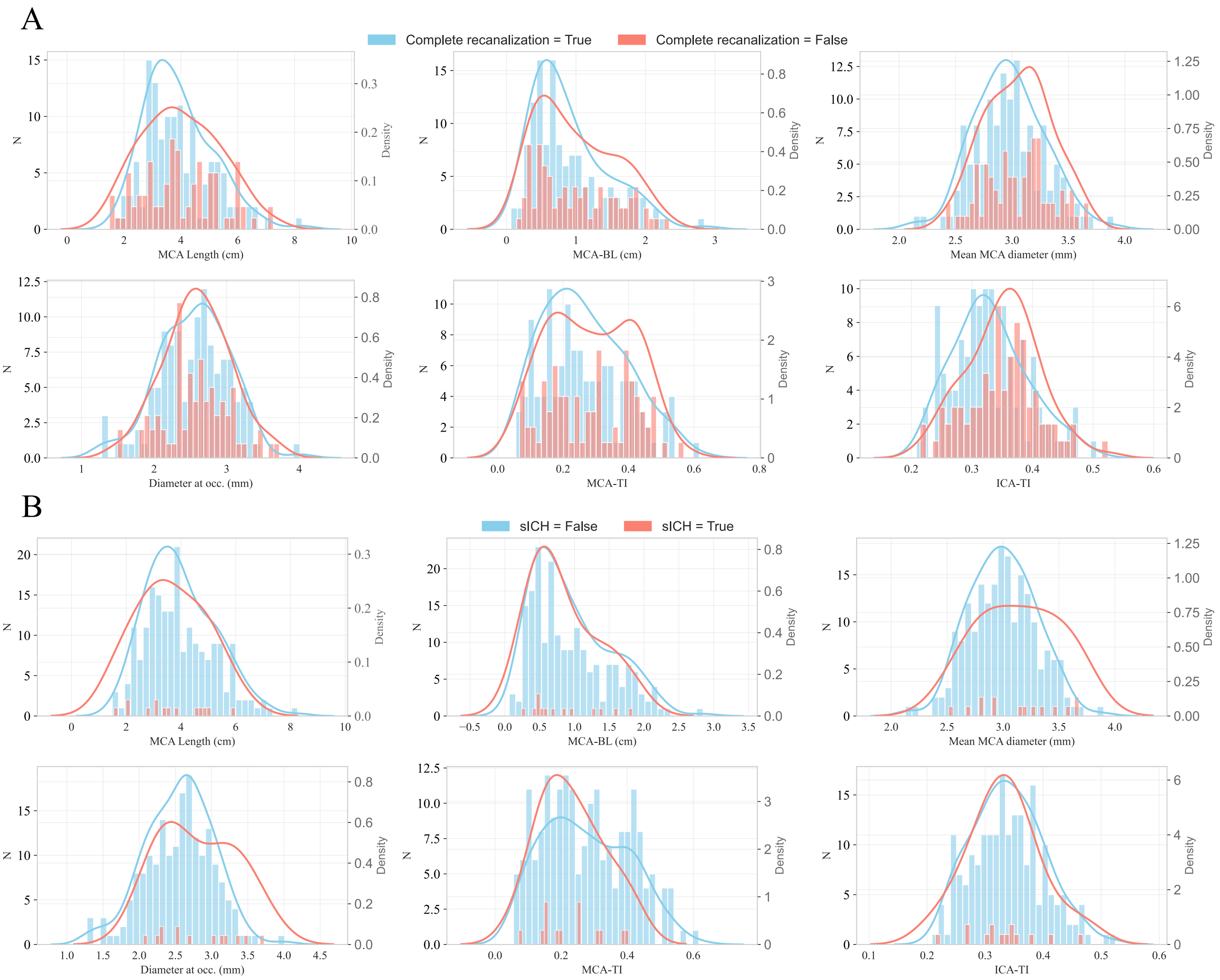

Figure A4. Histograms (left axis, bars) and KDE plots (right axis, continuous line) showing feature distributions across primary endpoints (A: complete recanalization; B: sICH). sICH: symptomatic intracranial hemorrhage. BL: bending length. MCA: middle cerebral artery. TI: tortuosity index. ICA: internal carotid artery.

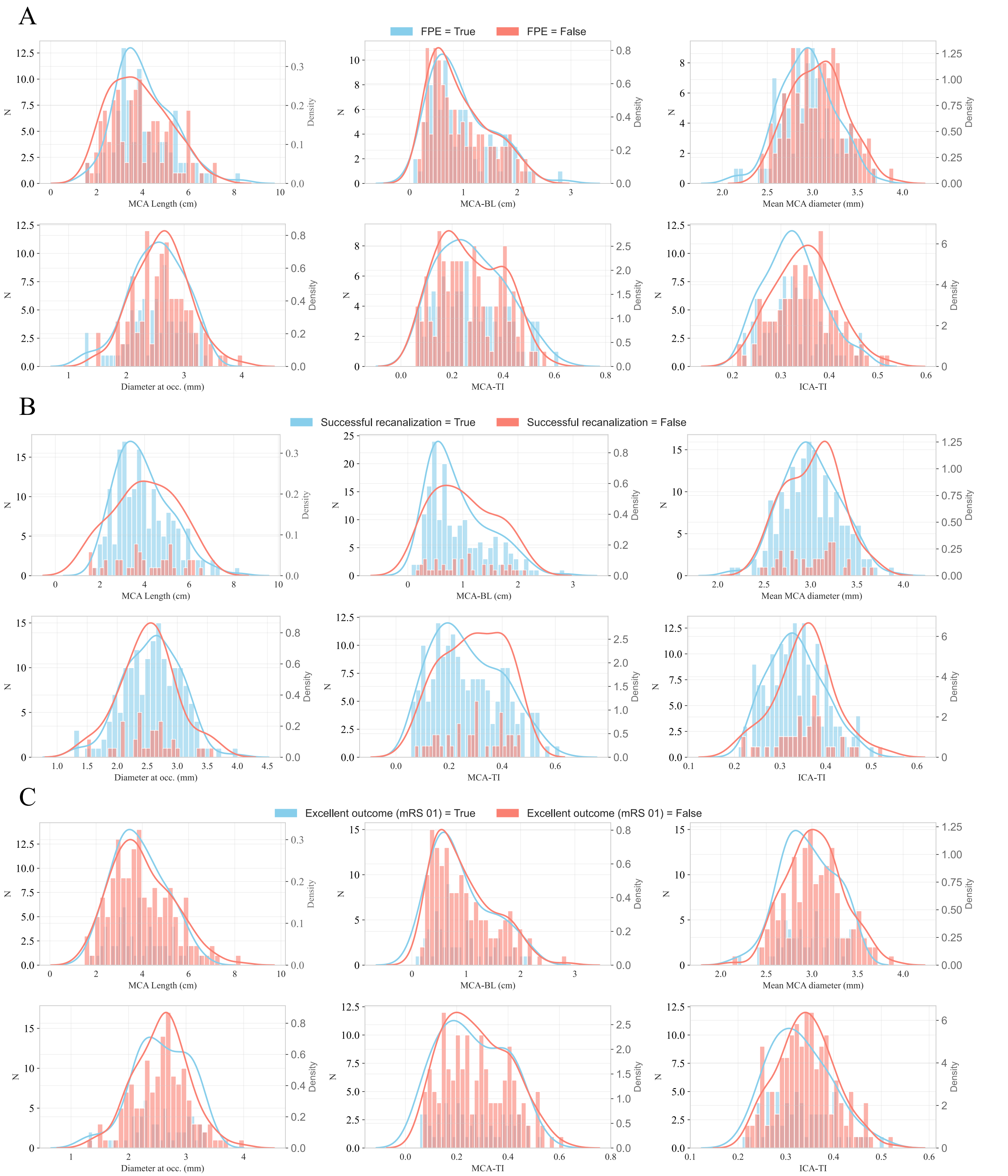

Figure A5. Histograms (left axis, bars) and KDE plots (right axis, continuous line) showing feature distributions across secondary efficacy endpoints (A: FPE; B: successful recanalization; C: excellent outcomes [mRS 0-1] at three months). BL: bending length. MCA: middle cerebral artery. TI: tortuosity index. ICA: internal carotid artery. FPE: first pass effect. mRS: modified Rankin Scale.

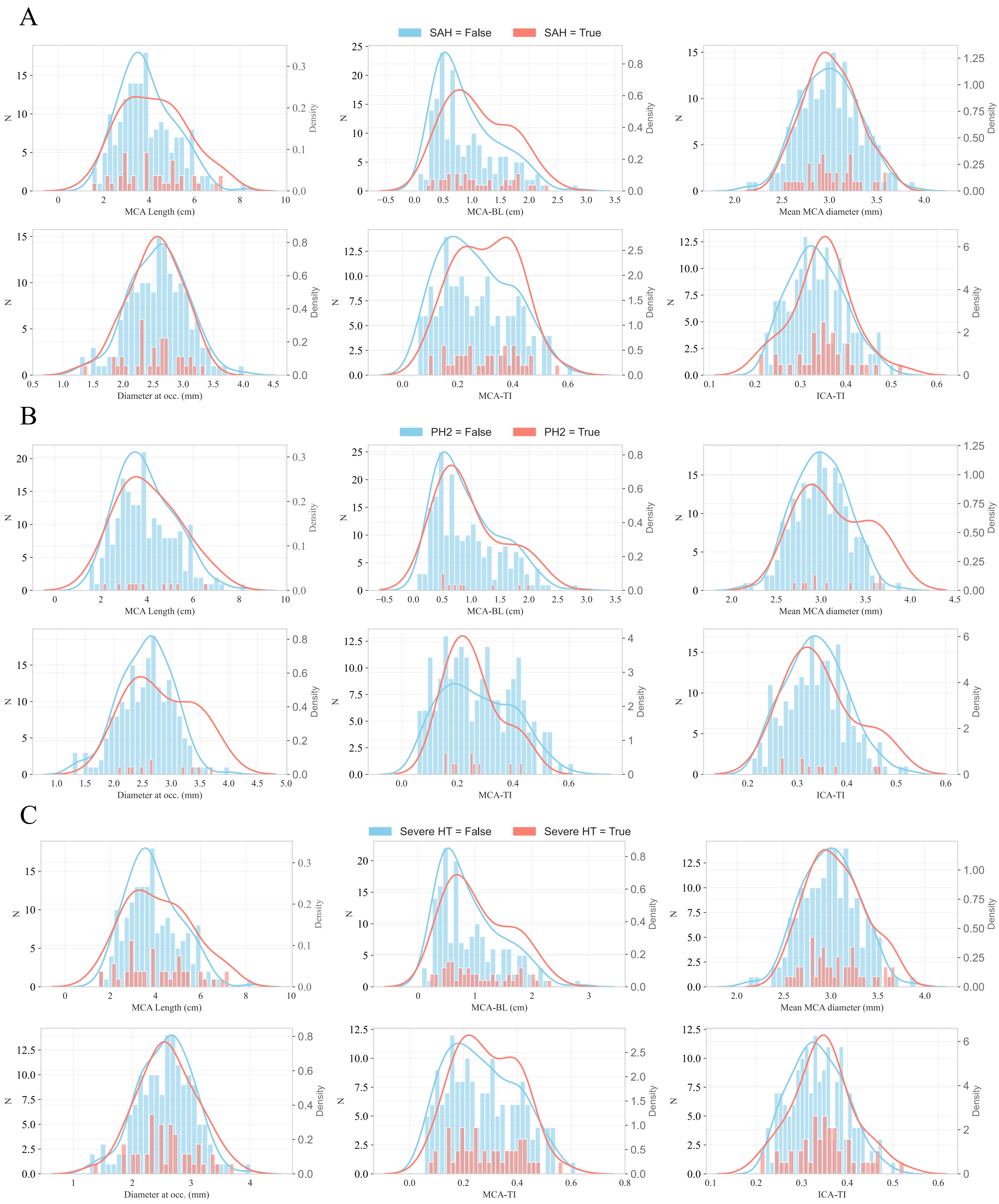

Figure A6. Histograms (left axis, bars) and KDE plots (right axis, continuous line) showing feature distributions across secondary safety endpoints (A: SAH; B: PH2; C: severe HT [SAH or PH2]). BL: bending length. MCA: middle cerebral artery. TI: tortuosity index. ICA: internal carotid artery. SAH: subarachnoid hemorrhage. PH2: parenchymal hematoma type 2. HT: hemorrhagic transformation.
